# Tuberculous Uveitis

**DOI:** 10.4103/0974-9233.58421

**Published:** 2009

**Authors:** Ahmed M. Abu El-Asrar, Marwan Abouammoh, Hani S. Al-Mezaine

**Affiliations:** Department of Ophthalmology, College of Medicine, King Saud University, Riyadh, Saudi Arabia

**Keywords:** Diagnosis, Treatment, Tuberculosis Uveitis

## Abstract

In recent years, ocular involvement due to TB has re-emerged. Tuberculous uveitis is a readily treatable disease and the consequences of delay in either ocular or systemic diagnosis can be very serious for the patient. It is important to have a high index of suspicion of the diagnosis in patients with unexplained chronic uveitis and this will be influenced by the socio-economic circumstances, family history, ethnic origin, and previous medical history of the patient. Treatment with antituberculous therapy combined with systemic corticosteroids resolves inflammation without recurrences after medical therapy.

## INTRODUCTION

Tuberculosis (TB) is re-emerging as a global health problem. It is a slowly progressive, chronic, granulomatous infection caused by *Mycobacterium tuberculosis*.^1^ This acid-fast bacillus usually affects the lungs, but can also affect other organs including the cardiovascular system, gastrointestinal system, musculoskeletal system, genitourinary tract, central nervous system, skin, and eyes.^2^,^3^

## EPIDEMIOLOGY OF TUBERCULOUS UVEITIS

Because of the lack of standardized diagnostic criteria for tuberculous uveitis, and the difficulty of confirming the diagnosis by laboratory methods, epidemiologic data for tuberculous uveitis are unreliable. By the 1940s, TB was considered to be the predominant cause of granulomatous uveitis. Guyton and Woods in 1941 assigned a tuberculous cause to 80% of granulomatous uveitis cases.^4^ However, this number steadily declined over subsequent decades. By 1960, Woods felt that only 20% of uveitis cases were secondary to TB.^4^Table 1 shows the frequency of tuberculous uveitis in the reported series of uveitis from different countries.^5^–^25^ Currently, tuberculous uveitis accounts for a relatively small number of cases in developed countries. However, a recent study from Japan by Wakabayashi *et al*.,^13^ showed an increasing frequency of tuberculous uveitis. Of 189 referred uveitis cases, 6.9% were found to have tuberculous uveitis. According to a study by Mercanti *et al*.,^15^ 7% of uveitis patients in Italy had presumed tuberculous etiology. In India, where pulmonary TB is endemic, TB was the cause of uveitis in 5.6^16^–10.1%^12^ of the cases. In a retrospective study of 200 referred patients with uveitis in Riyadh, Saudi Arabia, Islam and Tabbara^14^ reported tuberculous etiology in 10.5% of cases. In a series of 301 patients admitted to King Abdullaziz University Hospital in Riyadh, Saudi Arabia with the diagnosis of panuveitis or posterior uveitis, 79 (26.2%) patients were diagnosed to have presumed tuberculous uveitis (Abu El-Asrar, *et al*., unpublished data). The high incidence of tuberculous uveitis in this selected series of complicated cases might be due to an increase in the number of expatriates from countries where TB is endemic, and a high index of suspicion of the diagnosis in patients with unexplained chronic uveitis. Another factor is that this is a tertiary referral center and only the complicated cases were admitted.

**Table 1:** Frequency of tuberculous uveitis in reported series from different countries

Author	Year	Country	Total no. of studied patients	No. of tuberculous uveitis (%)
Kazokoglu *et al*.^5^	2008	Turkey	761	3 (0.3)
Pathanapitoon *et al*.^6^	2008	Thailand	200	3 (2.2)
Khairallah *et al*.^7^	2007	Tunisia	472	5 (1.1)
Rathinam and Namperumalsamy^8^	2007	India	8759	488 (5.6)
Yang *et al*.^9^	2005	China	1752	13 (0.7)
Sengun *et al*.^10^	2005	Turkey	300	4 (1.3)
Soheilian *et al*.^11^	2004	Iran	544	8 (1.5)
Singh *et al*.^12^	2004	India	1233	125 (10.1)
Wakabayashi *et al*.^13^	2003	Japan	189	13 (6.9)
Islam and Tabbara^14^	2002	Saudi Arabia	200	21 (10.5)
Mercanti *et al*.^15^	2001	Italy	655	46 (7.02)
Kaimbo Wa Kimbo *et al*.^16^	1998	Congo	336	20 (6)
Kotake *et al*.^17^	1997	Japan	551	1 (0.2)
Merril *et al*.^18^	1997	United States	385	2 (0.5)
Rodriguez *et al*.^19^	1996	United States	1273	8 (0.6)
Thean *et al*.^20^	1996	United Kingdom	712	2 (0.28)
Smit *et al*.^21^	1993	Netherland	750	20 (2.7)
Rothova *et al*.^22^	1992	Netherland	865	12 (1.4)
Weiner and Ben Ezra^23^	1991	Israel	400	3 (0.7)
Palmares *et al*.^24^	1990	Portugal	450	10 (2.2)
Henderly *et al*.^25^	1987	United States	600	1 (0.2)

## CLINICAL FEATURES

**Figure 1:**
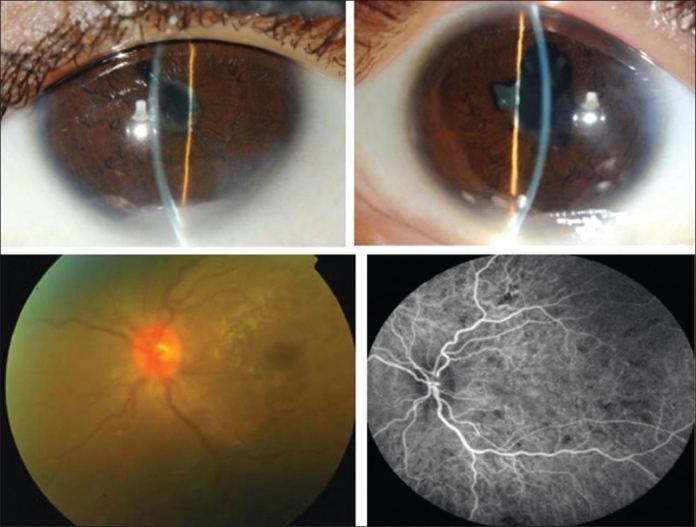
Slit-lamp biomicroscopy of a 45-year-old woman with strongly positive tuberculin skin test (22 mm induration) shows mutton-fat keratic precipitates, posterior synechiae and anterior chamber fibrinous exudate (top). Fundus photograph shows optic disc swelling and hyperemia (bottom left). Indocyanine green angiography shows choroidal hypofluorescent areas (bottom left)

**Figure 2:**
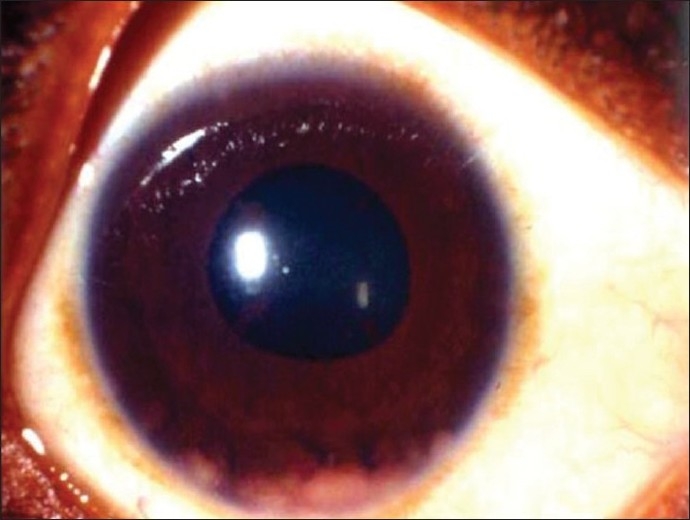
Right eye of a 21-year-old woman with strongly positive tuberculin skin test (22 mm induration) and a family history of tuberculosis shows large iris granulomas in the inferior angle

**Figure 3:**
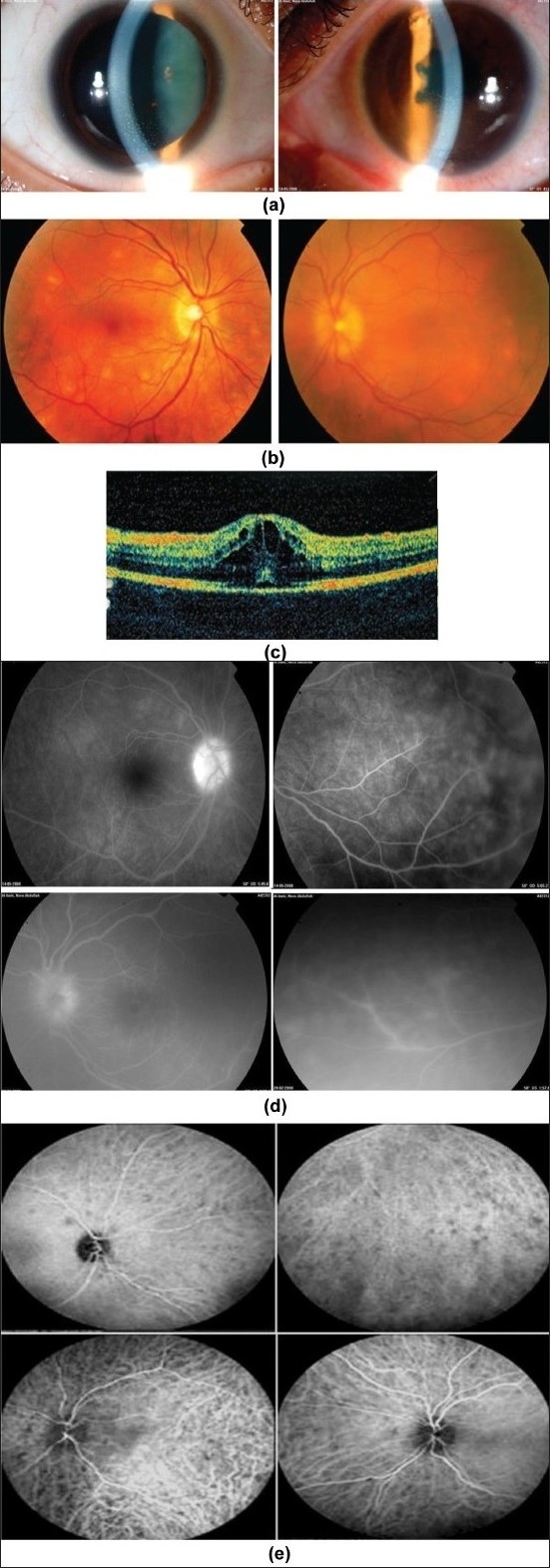
A 25-year-old woman with strongly positive tuberculin skin test (15 mm induration). (a) Slit-lamp biomicroscopy shows bilateral mutton-fat keratic precipitates and posterior synechiae in the left eye; (b) Fundus photographs show yellow lesions and disc swelling and hyperemia; (c) Optical coherence tomography of the left eye shows cystoid macular edema (d) Fluorescein angiography shows leakage from optic nerve head and retinal vessels; (e) Indocyanine green angiography shows choroidal hypofluorescent areas

**Figure 4:**
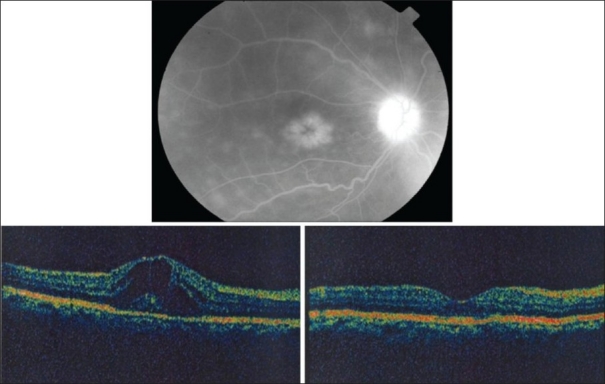
Right eye of a 45-year-old woman with strongly positive tuberculin skin test (20 mm induration). Fluorescein angiography shows leakage from optic nerve head and cystoid macular edema (top). Optical coherence tomogrqaphy shows cystoid macular edema. Central macular thickness was 588 μm. Visual acuity was 20/100 (bottom left). Two months after starting antituberculous therapy and systemic corticosteroids, optical coherence tomography displays normal anatomy of the macula with reduction of central macular thickness to 239 μm. Visual acuity improved to 20/30 (bottom right)

**Figure 5:**
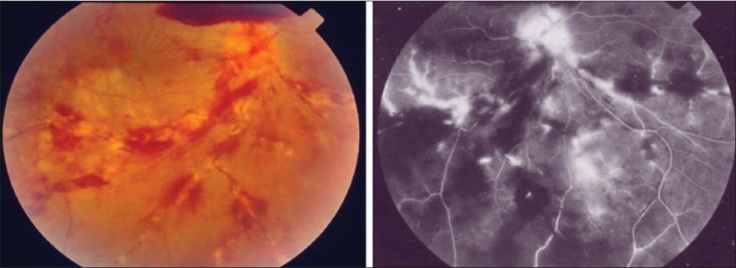
The right eye of a 28-year-old man with strongly positive tuberculin skin test (20 mm induration) shows thick perivenous sheathing with intraretinal hemorrhages, cotton-wool spots, neovessels on optic nerve head, and preretinal hemorrhage above optic nerve head (left). Fluorescein angiography shows leakage from the retinal veins, and neovessels on optic nerve head and retinal nonperfusion (right)

**Figure 6:**
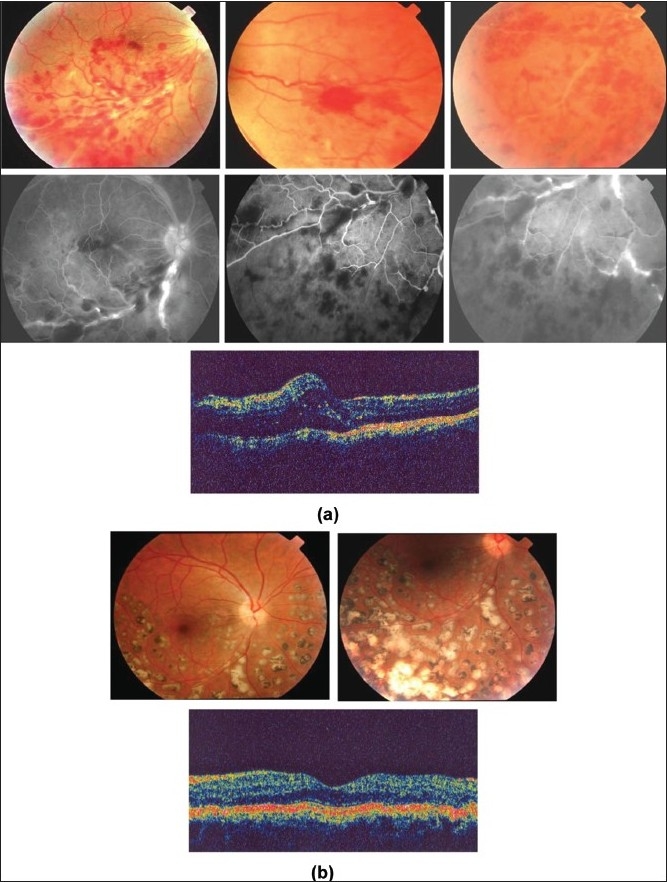
(a) Right eye of a 25-year-old man with strongly positive tuberculin skin test (16 mm induration) shows perivenous sheathing with intraretinal hemorrhages and neovessels nasal to optic nerve head (top). Fluorescein angiography shows leakage from the retinal veins and retinal nonperfusion (middle). Optical coherence tomography shows macular edema (bottom); (b) Ten months after treatment with antituberculous therapy, systemic corticosteroids, and scatter laser photocoagulation. Optical coherence tomography displays normal anatomy of the macula

**Figure 7:**
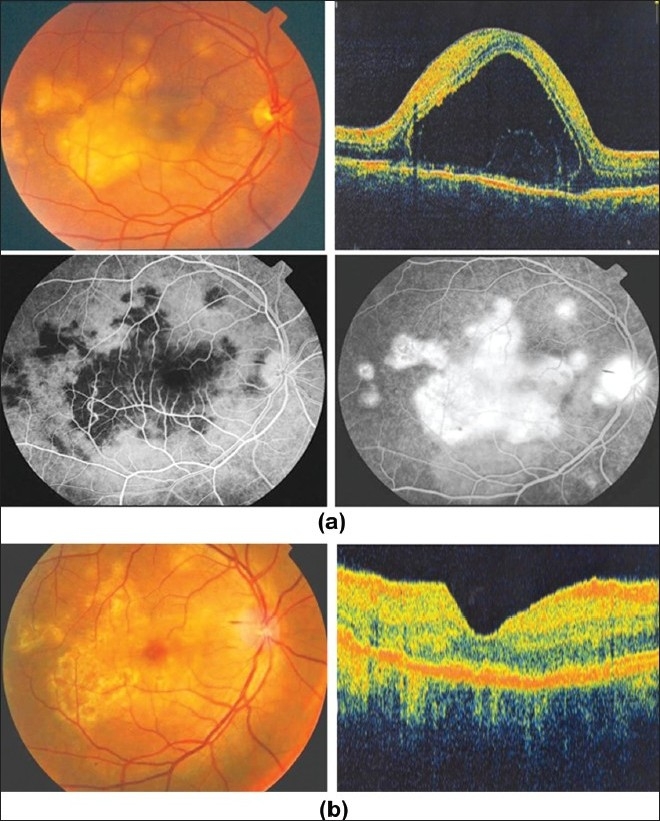
(a) Right eye of a 33-year-old man with strongly positive tuberculin skin test (24 mm induration) shows multifocal choroiditis. Visual acuity was 20/200 (top left). Optical coherence tomography shows overlying exudative retinal detachment (top right). Fluorescein angiography shows hypofluorescence in the early phase with staining in the late phase (bottom); (b) Nine months after starting antituberculous therapy and systemic corticosteroids. Optical coherence tomography displays resolution of exudative retinal detachment. Visual acuity improved to 20/20

**Figure 8:**
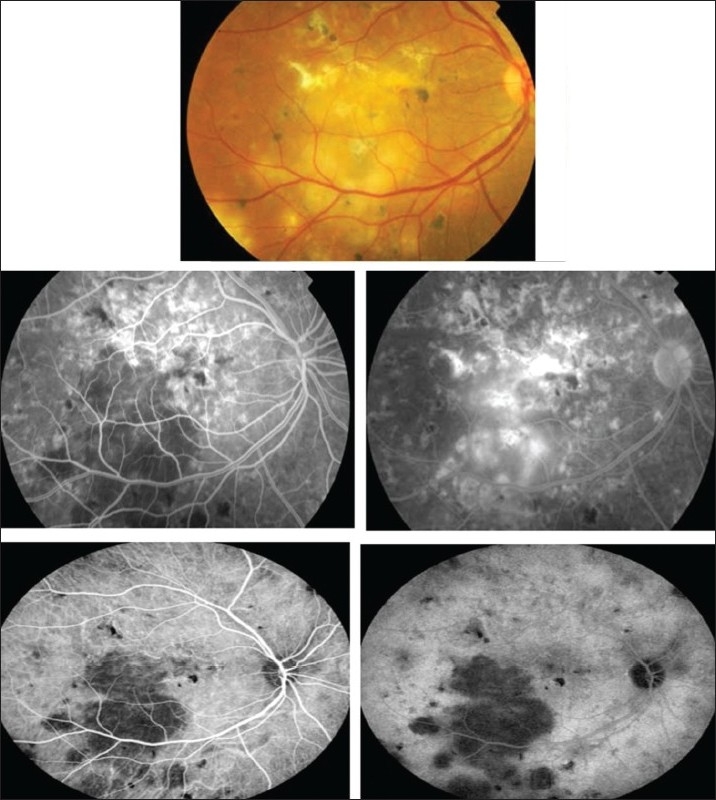
Right eye of a 31-year-old woman with strongly positive tuberculin skin test (18 mm induration) shows multifocal choroiditis (top). Fluorescein angiography shows early hypofluorescence and late hyperfluorescence (middle). Indocyanine green angiography shows hypofluorescent lesions throughout (bottom)

**Figure 9:**
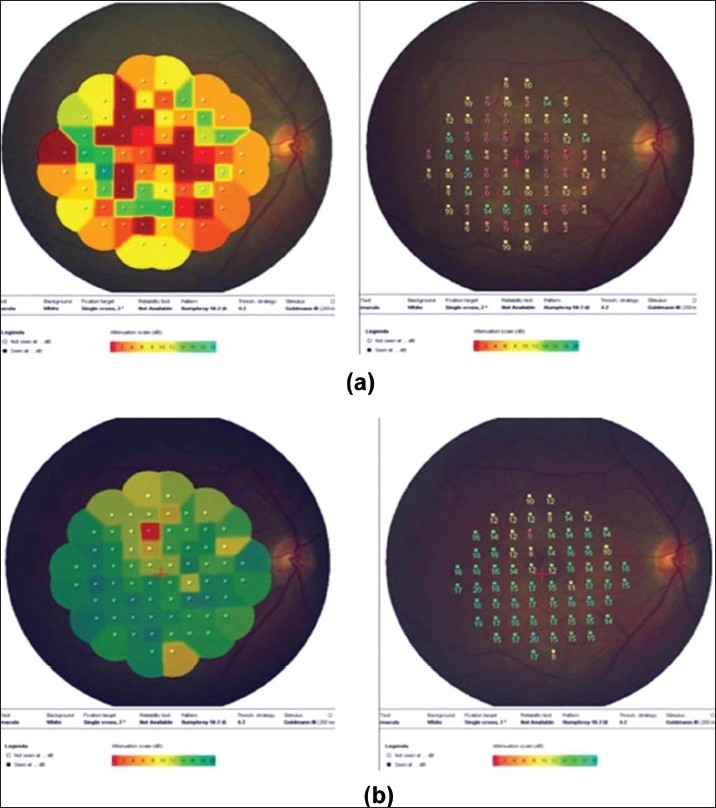
(a) Right eye of a 54-year-old man with strongly positive tuberculin skin test (22 mm induration) shows multifocal choroiditis. Visual acuity was 20/60. Fundus microperimetry shows decreased sensitivity (The local sensitivity of the measured points is shown in two ways-first as a colour code with dark green for best sensitivity and second as a numerical code in dB from 0 to 20 with 20 meaning best sensitivity. (b) Six months after starting antituberculous therapy and systemic corticosteroids. Visual acuity improved to 20/20 and microperimetry shows improved sensitivity

**Figure 10:**
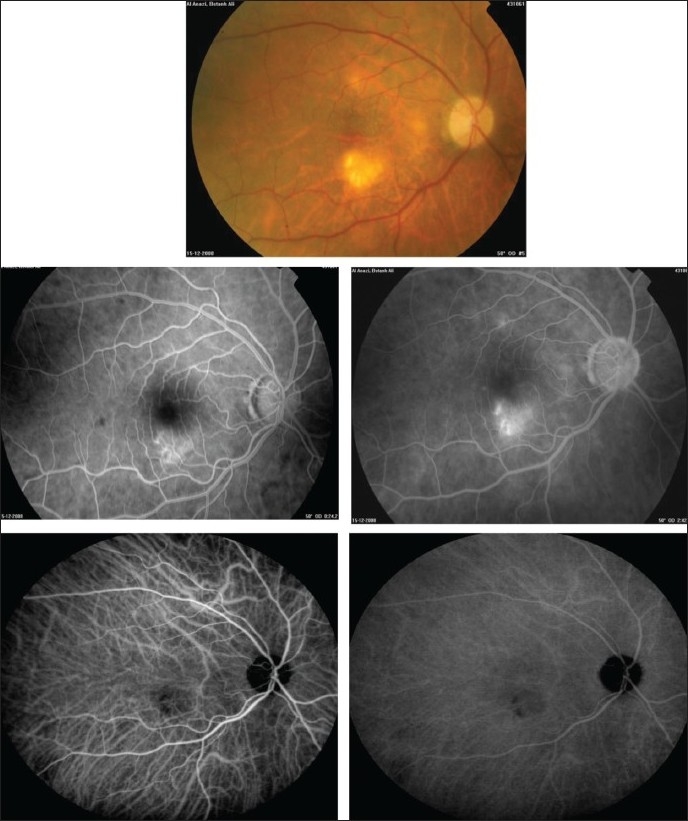
Right eye of a 60-year-old woman with strongly positive tuberculin skin test (30 mm induration) shows choroidal granulomas (top). Fluorescein angiography shows early hyperfluorescence and increase in hyperfluorescence during the late phase (middle). Indocyanine green angiography shows hypofluorescence throughout corresponding to the large granuloma (bottom)

Clinically, intraocular TB can be due to direct infection or indirect immune-mediated hypersensitivity response to mycobacterial antigens when there is no defined active systemic lesion elsewhere or the lesion is thought to be inactive^26^–^30^ [Table 2]. Intraocular TB is a great mimicker of various uveitis entities. The clinical manifestations of intraocular TB include acute anterior uveitis, chronic granulomatous anterior uveitis which may be associated with iris or angle granulomas, mutton-fat keratic precipitates and posterior synechiae [Figures 1–3], intermediate uveitis, vitritis, macular edema [Figures 3 and 4], retinal vasculitis [Figures 5 and 6], neuroretinitis, solitary or multiple choroidal tubercles, multifocal choroiditis [Figures 7–9], choroidal granulomas [Figure 10], subretinal abscess, endophthalmitis, and panophthalmitis.^26^–^44^

**Table 2:** Possible pathogenesis of tuberculous uveitis

Sign	Possible pathogenesis
Iris or angle granulomas	Direct infection
Ciliary body tubercle	Direct infection
Retinal vasculitis	Immune-mediated
Choroiditis	Immune-mediated
Choroidal tubercle	Direct infection
Subretinal abscess	Direct infection
Endophthalmitis	Direct infection
Panophthalmitis	Direct infection

Choroidal tubercles and tuberculomas (large, solitary masses) are reported to be the most common intraocular manifestations of TB. The presence of choroidal tubercles is indicative of hematogenous seeding of bacilli.^27^ There may be an overlying exudative retinal detachment. The differential diagnosis includes sarcoid granulomata, syphilitic gummas, and metastatic tumors. Choroidal TB might also present as multifocal progressive choroiditis, that shows progression to confluent, diffuse choroiditis wi th an active edge or diffuse choroiditis with amoeboid pattern and a leading edge. These morphologic presentations resemble serpiginous choroiditis.^34^,^38^

Tuberculous retinal vasculitis is typically an obliterative periphlebitis affecting the retina in multiple quadrants, starting at or anterior to the equator and progressing posteriorly. Occasionally, it can begin close to the optic nerve head, mimicking a vein occlusion. Ophthalmoscopic findings vary and depend on the stage of the disease. Initially, it presents as active retinal periphlebitis with thick exudates around the retinal veins associated with retinal hemorrhages, and hemorrhagic infarction of the retina. Active retinal periphlebitis is associated with mild degree of cellular infiltrate in the anterior chamber and mild vitreous infiltrate. Healed periphlebitis results in sclerosed venules and abnormal vascular anastomosis. The periphlebitis may cause nonperfusion of a substantial portion of the retina that may lead to proliferative vascular retinopathy with sequelae such as recurrent vitreous hemorrhage, traction retinal detachment, rubeosis iridis, and neovascular glaucoma [Figures 5 and 6].^26^,^31^,^36^

Of these various intraocular changes, the most common clinical presentation appears to be posterior uveitis. In a series of 158 patients with presumed intraocular TB from India, 66 (42%) had posterior uveitis, 57 (36%) anterior uveitis, 18 (11%) panuveitis, and the remaining 17 (11%) intermediate uveitis.^39^ In our series of 51 patients (73 eyes) with presumed tuberculous uveitis from Saudi Arabia, 58 (79.5%) eyes had panueveitis, and 15 (20.5%) eyes had posterior uveitis at presentation. Clinical manifestations included vitritis in 52 (71.2%) eyes, macular edema in 46 (63%) eyes, retinal periphlebitis in 26 (35.6%) eyes, multifocal choroiditis in 15 (20.5%) eyes, and granulomatous anterior uveitis in 13 (17.9%) eyes.^43^ In a series of 37 patients with the clinical diagnosis of presumed tuberculous uveitis followed at Italian and Swiss Centres, 26 (70.3%) patients presented with granulomatous panueveitis, 8 (21.6%) posterior uveitis, 2 (5.4%) nongranulomatous uveitis with synechiae and iris infiltration.^40^

## DIAGNOSIS

In recent years, ocular involvement due to TB has re-emerged associated with an increasing prevalence of TB. A high index of clinical suspicion is essential for the early diagnosis of tuberculous uveitis. Late diagnosis and delay in management can result in loss of the eye and can even be life-threatening in severe conditions. The diagnosis should be considered by the ophthalmologist when unexplained chronic uveitis with the characteristic clinical signs occurs that promptly recurs upon tapering corticosteroid and/or immunosuppressive therapy. Tuberculous retinal vasculitis should be suspected in the presence of florid retinal periphlebitis with marked capillary closure with a relatively mild degree of vitreous cellular infiltrate, particularly in patients of Asiatic origin: Genetic predisposition may account for the propensity to develop retinal vasculitis in these patients.^26^ Most patients with ocular involvement have no history of pulmonary or other systemic forms^26^,^27^,^35^,^40^,^43^ making a definitive diagnosis difficult. Therefore, tuberculous uveitis is frequently misdiagnosed and the disease is recognizable after a very long diagnostic delay.^40^ The absence of clinically evident pulmonary TB does not rule out the possibility of ocular TB, as about 60% of patients with extrapulmonary TB have no evidence of pulmonary TB.^45^

The diagnosis of ocular involvement with TB is considered in the setting of (1) isolation of *M*. *tuberculosis* from ocular fluid or tissue specimen by a microbiologic or histopathologic study. However, the process is prolonged as it may take several weeks before culture results become available for starting specific therapy. Moreover, obtaining biopsy specimens from intraocular tissues for making a confirmatory histopathological diagnosis is difficult with potential morbidity associated with obtaining the biopsy material from the eye; (2) as presumed ocular disease suggestive of TB with proven active systemic disease; or (3) as presumed ocular disease without evidence of active systemic disease.^26^ In the last two situations, the diagnosis of ocular TB remain largely presumptive. Because of the difficulty in obtaining microbiologic evidence, in nearly all reported cases, the diagnosis of intraocular TB was only presumptive.^26^,^29^,^30^,^32^,^37^,^40^,^43^

In most studies, the diagnostic criteria for presumed tuberculous uveitis were.^26^,^29^,^30^,^32^,^37^,^40^,^43^

1. Ocular findings consistent with possible intraocular TB with no other cause of uveitis suggested by history of symptoms, or ancillary testing.
2. Strongly positive tuberculin skin test results (≥15 mm area of induration/necrosis).
3. Response to antituberculous therapy with absence of recurrences.

### Tuberculin skin testing

Tuberculin skin test is of great importance for making the diagnosis of ocular TB. The standard test consists of an intradermal injection of 5 tuberculin units to raise a wheal of 6–10 mm in diameter. After 48 to 72 h, any induration is measured in millimeters transversely on the skin at the point of injection. An induration of less than 5 mm is considered to be a negative result. An induration of 5–10 mm is considered to be positive in patients with HIV infection, immunosuppressed patients, in those who are in close contact with a patient with active TB and those showing healed TB lesions on chest radiography. An induration of more than 10 mm is considered to be positive for those patients living in high endemic areas of TB and for employees of high risk congregate settings (e.g., health care workers). An induration of more than 15 mm is considered to be positive in all patients. Overwhelming TB illness, Hodgkin's disease, sarcoidosis, uremia, aging, corticosteroid use, and viral illness including HIV infection may be associated with false-negative reactions.

The primary use of the tuberculin skin test is to provide supportive information when clinical signs and symptoms suggest TB. When interpreting the result of a tuberculin skin test, one should consider the size of the reaction, contact history, regional prevalence of atypical mycobacteria, and patient characteristics such as age and immune status. The specificity of the tuberculin skin test for *M*. *tuberculosis* increases with larger skin reactions and with a history of exposure to an active case of TB.^27^ It is important to note that the effect of neonatal vaccination with bacilli almette-Guérin (BCG) on tuberculin skin test declines over the first seven years of life. In addition, it was demonstrated that an induration greater than 14 mm is unlikely to be due to prior BCG vaccination.^46^ In our series of 51 patients with presumed tuberculous uveitis, the mean area of induration was 25.2 ± 11.9 mm (range, 15–80 mm).^43^ Until tests with greater proven specificity and sensitivity are developed, we believe that tuberculin skin test should remain an integral part of systemic work-up for uveitis patients.

### Radiology

In patients with suspected tuberculous uveitis, chest X-ray and/or computed tomography of the chest may aid in the evaluation. Computed chest tomography was found to reveal the presence, dimensions, and activity of tuberculous mediastinal lymphadenopathy which routine chest X-rays were unable to detect in patients with presumed tuberculous uveitis.^47^

### Interferon-gamma release assays

Recent advances in mycobacterial genomics and human cellular immunology have resulted in two new blood tests that detect TB infection by measuring *in vitro t*-cell interferon (IFN)-gamma release in response to two unique antigens that are highly specific for *M*. *tuberculosis* but absent from BCG vaccine and most nontuberculous mycobacteria. One assay, the enzyme-linked immunospot (ELISpot) [T-SPOT.TB; Oxford Immunotec; Oxford, UK] enumerates IFN-gamma-secreting T cells, while the other assay measures IFN-gamma concentration in supernatant by enzyme-linked immunosorbent assay (ELISA) [QuantiFERON-TB Gold, QuantiFERON-TB Gold In-Tube; Cellestis; Carnegie, Australia]. A large and growing clinical evidence base indicates that both tests are more specific than the skin test because they are not confounded by prior BCG vaccination. In active tuberculosis, ELISA has similar sensitivity to the skin test, while ELISpot is significantly more sensitive. Current cross-sectional evidence suggests that for diagnosis of latent tuberculosis infection, sensitivity of ELISA is similar to tuberculin skin test, while ELISpot appears more sensitive.^48^,^49^ However, in a cross-sectional comparison study, Mazurek *et al*.,^50^ demonstrated that there was no statistically significant difference in specificity between the QuantiFERON-TB Gold assay and tuberculin skin test and that QuantiFERON-TB Gold assay may be less sensitive than the tuberculin skin test using a 15-mm induration cutoff value. Pollock *et al*.^51^ demonstrated extreme discordance between the results of their clinical diagnostic algorithm and the results of QuantiFERON-TB Gold assay and raised concern about the sensitivity of QuantiFERON-TB Gold assay for detection of latent TB infection in their newly hired healthcare workers. Moreover, in individuals with active TB, QuantiFERON-TB Gold assay was less sensitive than the tuberculin skin test.^52^ In 12 patients with granulomatous intraocular inflammatory disease, Kurup *et al*.,^53^ reported no demonstrable advantage of QuantiFERON-TB test over tuberculin skin test for detection of latent TB infection.

### Molecular techniques

Cultures of *M*. *tuberculosis* on Löwenstein-Jenson (egg-based) medium require at least four to eight weeks. The visible colonies are identified by Ziehl-Neelsen Stain. However, the process is prolonged, and it may not provide positive results because of the lower yield of organisms from the intraocular fluids. In recent years, molecular techniques are used for the diagnosis of TB. These techniques include polymerase chain reaction (PCR) to detect mycobacterial DNA in clinical specimens, nucleic acid probes to identify culture, restriction fragment length polymorphism analysis to compare strains for epidemiologic purposes, and genetic-base susceptibility testing methods for rapid detection of drug resistance.^54^ The false-positive PCR results and the low specificity of PCR might challenge the understanding of PCR results.^55^ PCR techniques were used for the detection of *M*. *tuberculosis* in aqueous and vitreous samples and epiretinal membranes from patients with presumed tuberculous uveitis,^31^,^33^–^35^,^56^,^57^ however, the sensitivity was reported to be low.^33^

### Imaging techniques

Intravenous fluorescein angiography is an essential component of the evaluation and management of presumed tuberculous retinal vasculitis. Characteristic features seen with fluorescein angiography in active vasculitis include diffuse leakage of dye due to breakdown of the inner blood-retinal barrier, and staining of the blood vessel with fluorescein. Fluorescein angiography is a more sensitive technique and will frequently show that the vasculitis is more extensive than the clinical examination suggests [Figures 5 and 6]. Fluorescein angiography is very useful to delineate areas of capillary nonperfusion, and neovascularization secondary to retinal ischemia [Figures 5 and 6]. The ability to identify retinal vasculitis as ischemic by fluorescein angiography has important implications for management and is discussed later. Other angiographic findings include cystoid macular edema and optic disc leakage [Figures 3 and 4]. Angiographically, active presumed tuberculous choroiditis shows early hypofluorescence and late hyperfluorescence [Figures 7 and 8].

Indocyanine green angiography (ICG) can detect subclinical choroidal involvement which is not detected by fundus examination or fluorescein angiography [Figures 1 and 3]. Wolfensberger *et al*.,^37^ identified four indocyanine green angiographic features. Hypofluorescent lesions were a constant finding that corresponded either to active lesions when they became isofluorescent or hyperfluorescent in the late phase (type 1 hypo-fluorescence) or to atrophic lesions when they remained hypofluorescent on indocyanine green, appearing as yellow lesions on ophthalmoscopy and hyperfluorescent areas on fluorescein angiography (type 2 hypofluorescence). Some of the lesions that remained hypofluorescent in the late phases of indocyanine green angiography responded to therapy, and probably corresponded to active granulomas occupying the full thickness of the choroid. Numerous focal hyperfluorescent spots were seen in eyes with more longstanding disease. Fuzzy choroidal vessels and late diffuse hyperfluorescence were signs of active disease and responded well to therapeutic intervention. These findings are not specific, although they represent a characteristic pattern of granulomatous choroiditis, such as ocular tuberculosis and sarcoidosis which represent very similar indocyanine green angiographic signs. In active choroiditis, the lesions are hypofluorescent during the early and the late phases of indocyanine green angiography [Figures 8 and 10].

Macular edema is a major cause of visual loss in patients with presumed tuberculous uveitis. In our series of 51 patients (73 eyes), macular edema was detected in 46 (63%) eyes. Optical coherence tomography (OCT) is the technique of choice to diagnose and for the follow-up and monitoring the effectiveness of treatment regimens on macular edema associated with presumed tuberculous uveitis^43^ [Figures 3,4 and 6]. OCT is also useful to delineate the extent of exudative retinal detachment overlying active choroiditis [Figure 7].

Ultrasonography is necessary to evaluate the status of the retina in eyes with dense vitreous hemorrhage complicating ischemic retinal vasculitis. It is also useful for those cases that present with choroidal tuberculoma masquerading as ocular tumors. It displays a lesion with low internal reflectivity and high vascularity on A-scan and with solid elevated mass with absence of scleral echo on B-scan.^44^ In addition, ultrasound biomicroscopy is a useful investigative tool to detect the presence of granuloma arising from the ciliary body.

The microperimeter-1 (MP-1; Nidek Technologies, Italy) is a new instrument that combines fundus tracking software, microperimetry, and color fundus photography. It uses scanning laser ophthalmoscope to evaluate retinal sensitivity, which is determined by means of a threshold strategy. MP-1 allows a retinal sensitivity map to be overlaid on a real-time fundus photograph, which makes it a good tool to assess retinal sensitivity in the macular area and to correlate the findings directly to the fundus location.^58^ Currently, we are using MP-1 to evaluate macular function and the efficacy of therapy in patients with presumed tuberculous choroiditis [Figure 9].

## TREATMENT

The treatment of ocular inflammation associated with TB should be directed against both the infection and the inflammatory reaction.^59^ Our recommended therapy for tuberculous uveitis consists of isoniazid 5 mg/kg/day, rifampicin 450 mg/day if body weight is < 50 kg and 600 mg if the weight is > 50 kg, ethambutol 15 mg/kg/day, and pyrazinamide 25 to 30 mg/kg/day initially for two months. Thereafter, rifampicin and isoniazid are used for at least another four to seven months. Oral prednisone is added at a dose of 1 mg/Kg/day until a clinical response is seen then a slow reduction is established. In our series of 51 patients (73 eyes) with presumed tuberculous uveitis, the duration of systemic corticosteroid therapy ranged from two to eight months with a mean of 4.2 ± 4.1 months and a median of 2.5 months. All eyes showed resolution of inflammation without any recurrence after stopping antituberculous therapy and systemic corticosteroids. In addition, this treatment regime induced a significant reduction in central macular thickness associated with a significant improvement in visual acuity in eyes with macular edema^43^ [Figure 4]. During treatment, patients should be monitored for drug toxicity. Therefore, the treatment should be instituted in consultation with an infectious disease specialist.

Previous studies reported a favorable response to antituberculous therapy when administered concomitantly with systemic corticosteroids in patients with presumed tuberculous uveitis.^26^,^31^,^32^,^34^,^38^,^41^,^43^ Rosen *et al*.,^26^ reported a series of patients with presumed intraocular TB in which one patient with retinal vasculitis associated with strongly positive tuberculin skin test result without evidence of active systemic disease at presentation developed miliary tuberculosis with choroidal tubercles following treatment with systemic corticosteroids alone. They strongly advocated the concomitant use of specific antituberculous therapy if presumed intraocular TB is suspected even in the absence of active systemic disease to avoid this complication. Moreover, several studies demonstrated that patients with presumed tuberculous uveitis who were treated only with systemic corticosteroids continued to have recurrent episodes of active inflammation or showed worsening and  inflammation was controlled only with concomitant treatment with antituberculous therapy.^31^–^34^,^40^,^41^ Although the inflammation can be controlled initially by the use of systemic corticosteroids alone, elimination of recurrences in patients treated with antituberculous drugs strongly favors the use of specific anti TB therapy in patients with presumed tuberculous uveitis.^31^–^34^,^40^,^41^ Antituberculous therapy in these patients could help by killing the intraocular microorganisms; thus resulting in reduced antigen load and resultant inflammation. The reduced antigen load would reduce the hypersensitivity reactions too, which probably results in eliminating the recurrences in these patients.^33^ Recently, Rao *et al*.,^60^ demonstrated selective distribution of *M*. *tuberculosis* in the retinal pigment epithelium of the enucleated eye of a case of panuveitis. Such findings suggest preferential localization of *M*. *tuberculosis* in the retinal pigment epithelium in eyes with panuveitis resulting from tuberculosis and also that recurrences in tuberculous choroiditis could result from reactivation of sequestered organisms in the retinal pigment epithelium. Prevention of such recurrences and elimination of the sequestered organisms require a longer course of systemic antimycobacterial agents, preferably at least six to nine months.^60^

The management of active retinal vasculitis requires the use of systemic corticosteroids and appropriate antituberculous therapy. New vessel formation associated with retinal vasculitis and capillary closure responds to panretinal photocoagulation. Early vitrectomy and adequate endolaser photocoagulation should be considered in eyes with nonresolving vitreous hemorrhage associated with active fibrovascular proliferation^36^ [Figure 6].
